# *Anisakis* sensitization in different population groups and public health impact: A systematic review

**DOI:** 10.1371/journal.pone.0203671

**Published:** 2018-09-20

**Authors:** Walter Mazzucco, Daniele Domenico Raia, Claudia Marotta, Antonella Costa, Vincenzo Ferrantelli, Francesco Vitale, Alessandra Casuccio

**Affiliations:** 1 Department of Science for Health Promotion and Mother to Child Care "G. D'Alessandro", University of Palermo, Palermo, Italy; 2 National Reference Center for Anisakiasis (C.Re.N.A.), Istituto Zooprofilattico Sperimentale della Sicilia “A. Mirri”, Palermo, Italy; Universita degli Studi di Roma La Sapienza, ITALY

## Abstract

*Anisakis simplex* spp. sensitization rates have increased worldwide, with a significant impact on health-care systems. To date, no clear-cut diagnostic criteria and laboratory algorithm have been established, so anisakiasis still represents an under-reported health problem whose clinical manifestations, when present, mimic the much more common allergic and digestive disorders. Aim of the study was to systematically review the available literature on the prevalence of sensitization against *Anisakis* in the general population and in specific population groups, taking into account the impact of the different available diagnostic techniques on the epidemiological data. Following the Preferred Reporting Items for Systematic Reviews and Meta-Analyses (PRISMA) statement, relevant papers reporting *Anisakis* sensitization epidemiological data were found covering a period ranging from 1996 to February 2017. Overall, 41 studies comprising 31,701 participants from eleven countries were included in the qualitative synthesis. General asymptomatic population resulted sensitized to *Anisakis* in 0.4 to 27.4% of cases detected by means of indirect ELISA or ImmunoCAP specific IgE detection, and between 6.6% and 19.6% of the samples by Skin prick test (SPT). Occupationally exposed workers (fishermen, fishmongers and workers of fish-processing industries) documented specific IgE between 11.7% and 50% of cases, whereas SPT positivity ranged between 8% and 46.4%. Symptomatic allergic patients to any kind of allergen were found to be positive to *Anisakis* specific IgE detection between 0.0% (in children with mastocytosis) to 81.3% (among adults with shellfish allergy). Results highlighted that hypersensitivity prevalence estimates varied widely according to geographical area, characteristics of the population studied, diagnostic criteria and laboratory assays. Further studies are needed to overcome the documented misdiagnosis by improving the diagnostic approach and, consequently, providing more affordable estimates in order to address public health interventions on populations at high risk of exposure to *Anisakis* and to tailor health services related to specific groups.

## Introduction

During the last decades, progress in the food industry and globalization have markedly increased the exposure to new allergenic sources that not always are adequately pointed out [1]. Coupled with changes in eating habits, including widespread consumption of raw, marinated or smoked fish, a quota of food allergies of unknown origin in the general population may be due to sensitization to *Anisakis* spp., representing a public health issue of growing importance [2,3]. Moreover, occupational contact was associated with *Anisakis* sensitization and allergic symptoms among fish-processing workers and fishmongers [4,5].

Humans can become accidental non-permissive hosts of the *Anisakis* parasite by eating parasitized raw or undercooked fish containing larvae in stage 3 [6,7]. Within hours after being ingested, *Anisakis* larvae penetrate the mucosal layers of the gastrointestinal tract, causing direct tissue damage that may lead to the zoonotic disease known as anisakiasis. This acute gastrointestinal form of *Anisakis* infection is usually transient, with the worm dying within a few weeks. It is manifested by clinical symptoms ranging from nausea, vomiting, diarrhoea, mild to severe abdominal pain and intestinal obstruction [8], mimicking other much more common gastrointestinal disturbances, such as acute appendicitis, gastric ulcer, or tumours, thus making diagnosis of anisakiasis extremely difficult.

Moreover, *Anisakis* is implicated in allergic IgE-mediated reactions, occurring after secondary exposure to the parasite, such as urticaria, angioedema, asthma and, rarely, anaphylaxis in highly sensitized people [2, 9–12]. Not by chance, in the past, allergic reactions to *Anisakis* have been mistaken for other entities such as acute urticaria or fish allergy [13]. Of interest, high levels of specific IgE for *Anisakis* allergens were also detected in healthy individuals without any clinical symptom.

The current diagnostic algorithm of *Anisakis*-related allergy has been based upon suggestive anamnesis (appearance of symptoms few hours after raw fish intake) along with positive skin prick testing, enzyme-linked immunosorbant assay (ELISA), ImmunoCAP or immunoblotting determination of antigen-specific IgE and exclusion of fish allergy, but the high number of false positives due to cross-reactivities with numerous panallergens has underlined the need to improve the diagnostic approach [14–17].

Often, these misdiagnosis lead to a domino cascade of useless diagnostic tests with significant healthcare costs [18].

The significant impact of *Anisakis* sensitization in the general population and in specific occupational settings (mainly allergic patients and fishing industry workers) has been stressed by several studies, particularly the ones with the largest sample size, held in Japan, Spain and Italy, documenting how *Anisakis* was a leading cause of food allergies more frequently than seafood itself [4,19–21]. Furthermore, sensitization to Anisakis was correlated not only with ingestion of contaminated fish, but also among workers whose occupation consisted of frequent handling of raw fish or fishmeal [4], also including cooks and restaurant workers [22–25].

The accurate assessment of *Anisakis* hypersensitivity prevalence plays a pivotal role to tailor health services and public initiatives according to the needs of the population, particularly in order to plan disease surveillance, ensure sufficient resources to cope with the burden of disease and evaluate trends over time [6]. Also, differences in diagnostic techniques and characteristics of populations enrolled led to conflicting reports among various geographical areas [8].

We performed a systematic review of the available literature on *Anisakis* sensitization prevalence in general population and other population strata, including occupationally exposed workers, taking into account the impact of the different available diagnostic techniques on the epidemiological data.

## Material and methods

### Search strategy

The Preferred Reporting Items for Systematic Reviews and Meta-Analyses (PRISMA)

Guidelines [26] were followed to conduct the systematic review of the literature **(S1 Table).** Ethics board review was not sought because this review used only publically available information.

#### Database search

A systematic review of peer-reviewed English-language literature for *Anisakis* spp. sensitization prevalence data was conducted through a search of Medline and Scopus databases. Initially, free text words representing broad concept of “*Anisakis* allergy” were used to identify the keywords (for example, Medical Subject Headings, MeSH) for subject searching. Then, a combination of MeSH terms and free text words were arranged in the following research string with OR and AND logical operators: *Anisakis AND (prevalence OR epidemiology) AND (allergy OR hypersensitivity OR immunization OR sensitivity OR sensitization OR ELISA OR skin prick test OR ImmunoCAP OR Immunoblot OR diagnostic techniques)*. Reference lists of the articles included in the analysis and of others relevant to the topic were hand-searched to identify additional potentially relevant publications, until no new information was found.

#### Other sources

Grey literature was identified by searching for conference or meeting abstracts and proceedings. The literature was last searched on February the 6^th^ 2017.

#### Inclusion and exclusion criteria

All articles meeting the following criteria were screened and then assessed for eligibility: peer reviewed manuscripts, published from 1996 to February 2017, reporting *Anisakis* sensitization prevalence estimates, a description of the population involved, the techniques used to test for immunization and the number of people tested.

Reports of analytical studies (cross-sectional studies, prospective or retrospective) were included, with no restriction on age or type of population. Review articles, conference abstracts, editorials and case reports were excluded.

#### Screening

After removal of duplicates, the records were screened by two reviewers (CM and DDR) in three levels. The first level included title screening, the second level included abstract screening and the third level included full text screening. For each level, the reviewers separately screened the records. Any disagreement was resolved by consensus with a third author (WM). After screening, studies were assessed for eligibility and final selection.

### Study quality assessment process

Quality assessment of thirty-seven studies included was performed by using an adapted version of the Joanna Briggs Institute Prevalence Critical Appraisal Tool [27], which was tailored to the objective and primary outcome measures of this review by modifying in order to account for specific *Anisakis* sensitization test criteria. Each study was assessed for ten criteria (**Table 1**): sample representativeness; participants recruitment; sample size; description of participants and setting; response rate; objective, reliable measurement of *Anisakis* sensitization; reliability of diagnostic techniques; appropriateness of statistical analysis, confounding factors/subgroups/differences identified and accounted for; identification of subpopulations using objective criteria (**S2 Table**). Being the maximum score obtainable equal to 14, a score of 7 was considered as cut off between middle-low and middle-high study quality.

**Table 1:** Criteria for the quality assessment of the studies (adapted from Joanna Briggs Institute Prevalence Critical Appraisal Tool).

Criteria	Score (Maximum score = 14)
**1.** Sample representativeness	Adequate = 1, Not Adequate = 0, NA
**2.** Participants recruitment	Random = 1, all other methods = 0, NA
**3.** Sample size	≥200 = 1, <200 = 0, NA
**4.** Description of participants and setting	Adequate = 1, Not Adequate = 0, NA
**5.** Response rate, %	<50% = 0, 50–80% = 1, >80% = 2
**6.** Objective, reliable measurement of *Anisakis* sensitization	3 diagnostic criteria (anamnestic, clinical, laboratoristic) = 3; only 2 up to 3 criteria = 2; only one criteria = 1
**7.** Reliability of diagnostic techniques	Antigens used specified in the text = 2; only anamnesis = 0, all the other measurement = 1
**8.** Appropriateness of statistical analysis	Adequate = 1, Not Adequate = 0, NA
**9.** Confounding factors/subgroups/differences identified and accounted for	Adequate = 1, Not Adequate = 0, NA
**10.** Identification of subpopulations using objective criteria	Adequate = 1, Not Adequate = 0, NA

When only the abstract was available, quality assessment could not be performed.

### Data extraction

Data were extracted using a data extraction MS Excel sheet. Data extraction included authors, year of publication, year of study, study location, study design, statistical measures, study settings, samples size, the characteristics of the studies’ participants, as age and females/males ratio, as well as diagnostic techniques and criteria employed to define *Anisakis* sensitization and allergy and relative prevalence estimates. DDR conducted data extraction, while CM performed the analysis of the studies’ quality.

### Study protocol

The study protocol has been registered with PROSPERO, number CRD42017057316.

A total of 248 records were identified searching in Medline and Scopus databases.

**Fig 1:**
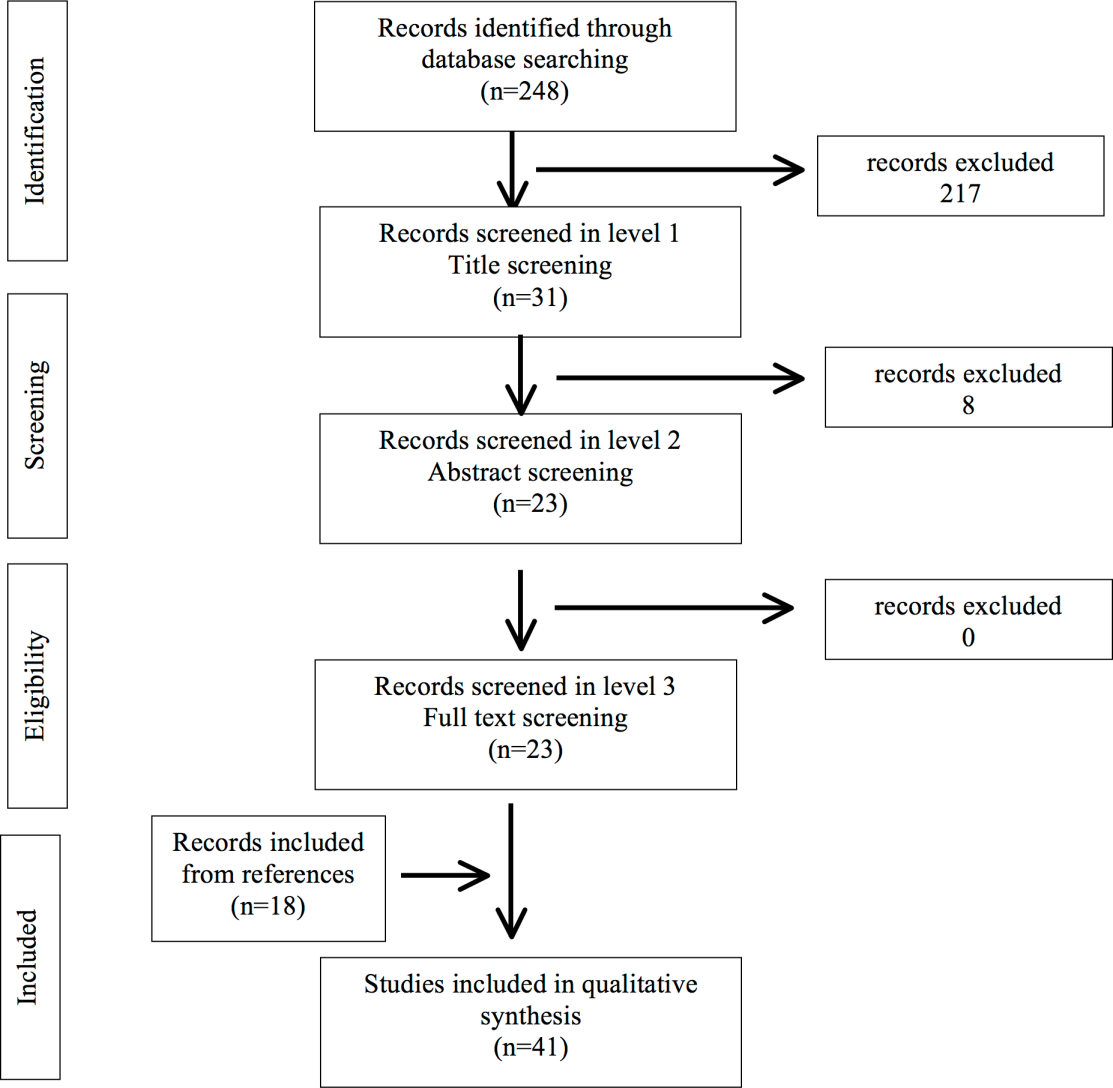
Flow diagram for selection of studies on *Anisakis* sensitization prevalence.

After title screening, 217 records were excluded. Of the remaining 31 manuscripts, 8 were removed subsequently to abstract evaluation. In the latest phase, full text assessment led to inclusion of all the remaining 23 manuscripts, and 18 more articles fulfilling the inclusion criteria found in the reference lists were added (**Fig 1**) (**S1 Database**).

### Diagnostic techniques employed

#### Skin prick test (SPT)

Sensitization to *Anisakis* is ruled out by the appearance of a >3 mm diameter wheal on the volar aspect of each subject's forearm after scratching the skin in the presence of a dilution of *Anisakis* extract (obtained by the centrifugation of total larvae in phosphate-buffered saline for 15 minutes at 1500 g) [28, 29].

#### ImmunoCAP, UniCAP-100, Radio-Allergo-Sorbent Test (RAST)

Total immunoglobulins (Igs), IgE, IgM, IgA1, IgG1, IgG4, antibodies against Anisakis crude extract, excretory-secretory antigens and recombinant antigens Ani s1, Ani s3, Ani s5, Ani s9, and Ani s10 are first detected by incubation with subjects’ serum samples and then revealed using an anti-human Igs labelled with radioactive or fluorescent marker [11, 30–33].

#### Indirect ELISA

Specific anti-Ani s1 and Ani s7 IgE antibodies are detected in patients’ sera after adding diluted *Anisakis* antigens to ELISA plates, and then incubating with secondary antibodies coupled to an enzyme. After washing, so that excess of unbound antibodies can be removed, a substrate is added, and remaining enzymes elicit a chromogenic or fluorescent signal, which is proportional to the antibody-antigen complexes and can be measured as optical density (OD) [34].

#### Antigen capture ELISA

It is a variation of indirect ELISA in which the serum to be tested is added to wells containing O-deglycosylated *A*. *simplex* antigen bound by the immobilized monoclonal antibody UA3, in order to detect specific IgG1 and IgE [35].

#### rAni s1, rAni s7 ELISA

Specific anti-Anisakis IgE antibodies are detected by indirect ELISA, with rAni s 7 or rAni s 1 as the target. After incubation of the plates and blocking of nonreactive sites, undiluted serum is added to each well and the specific IgE detected [36].

#### Immunoblotting

*Anisakis* specific IgE detection is performed by means of sodium dodecylsulfate-polyacrylamide gel electrophoresis with a dilution of *Anisakis* extract or recombinant Ani s1, Ani s3, Ani s5, Ani s9 and Ani s10. Proteins are afterwards transferred to nitrocellulose membranes and incubated overnight with diluted sera from patients in incubation buffer. After washing, the membranes are incubated with appropriate dilution of monoclonal labelled antihuman immunoglobulins [19, 37].

#### Basophil activation test (BAT)

Flow-cytometry expression of CD63 on activated basophils is measured on whole blood sample after incubation in a water bath with *Anisakis* crude extract. Samples are lysed, washed and re-suspended to be measured in a flow cytometer after staining cells with 20 μL of CD63-FITC ⁄ CD123-PE ⁄ anti-HLA-DR-PerCP reagent mixture cocktail. Activated basophils were finally identified using anti-CD123, anti-HLA-DR and anti-CD63 monoclonal antibodies [38].

## Results

The characteristics of the 41 studies reviewed in the qualitative analysis are summarized in **Table 2**.

**Table 2:** Characteristics of the n. 41 studies included in the systematic review.

Author, year	Country	Study design	Study period	Study setting	Study participants	N° of participants	Age mean (SD) or median (IQR), range	Female n/N (%)
**Abattouy, 2013**	Morocco	Cross sectional	Not provided	Public clinical analysis laboratories and general practitioner	Inhabitants and fishing industry workers from 2 coastal cities	333	38.4 22–70	156/333 (46.8)
**Anadon, 2010**	Spain	Cross sectional	1995–2001	Allergy service of Madrid hospital	Serum samples of Madrid allergic inhabitants	495	44.3 5–81	322/495) (65)
**Andreu-Ballester, 2008**	Spain	Case-control	Not provided	1 hospital	Appendectomized patients and non appendectomized patients who presented to the emergency department	160 (80 appendectomized patients and 80 non appendectomized patients)	39.2 (14) 20–75	76/180 (47.5)
**Añíbarro, 2007**	Spain	Cross-sectional	Not provided	1 allergy unit covering 400.000 inhabitants public health area	Food allergic patients	436	46 (13.8) (mean age onset of symptoms)	Not provided
**Asero, 2009**	Italy	Cross-sectional	2007	19 allergy outpatient clinics	Food allergic patients	1,110	31 12–79	719/1,110 (64.7)
**Bernardini, 2000**	Italy	Cross-sectional	Not provided	1 allergy unit	Children with allergic symptoms	805	7.9 (3.8) 0.52±17.61	324/805 (40.2)
**Caballero,** **2012**	Spain	Cross-sectional	Not provided	1 allergy unit	Tolerant patients with suspected not-fish-related allergy and patients with allergy to *A*. *simplex*.	99 (tolerant patients with suspected not-fish-related allergy) 35 (patients with allergy to *A*. *simplex)*	36.2 (tolerant patients with suspected not-fish-related allergy) 52.5 (patients with allergy to A. simplex).	Not provided
**Consortium AAITO-IFIACI** **Anisakis, 2010**	Italy	Cross-sectional	2010	34 allergy units	Suspected allergy patients	10,570	Not provided	Not provided
**Daschner, 2005**	Spain	Cross-sectional	Not provided	1 allergy unit	Chronic urticaria patients	135	41.5 (15.4)	91/135 (67.4)
**del Pozo, 1997**	Spain	Cross-sectional	Not provided	1 allergy unit	Urticaria/angioedema patients	100	37.4 18–75	63/100 (63.0)
**Del Rey Moreno, 2006**	Spain	Cross-sectional	2000	1 hospital laboratory	Random healthy blood donors	77	Not provided	Not provided
**Estrada Rodriguez, 1997**	Spain	Cross-sectional	Not provided	1 hospital laboratory	Not provided	66	Not provided	Not provided
**Falcao, 2008**	Portugal	Case-control	Not provided	Immuno- allergology and surgery units of the largest paediatric hospital in Porto	Cases with of acute urticaria Controls among programmed surgery patients	200 (cases with of acute urticarial). 200 (controls among programmed surgery patients). 400 overall	6–18	66/200 (33.0) for cases. 92/200 (46.0) for controls. Overall 158/400 (39.5)
**Figueiredo, 2013**	Brazil	Cross-sectional	2010	1 military facility	Healthy adult affiliated with a military facility	67	40 ± 8.4 years (median)	Not provided
**Figueiredo, 2015**	Brazil	Cross-sectional	2009–2010	2 perinatal centers (1 high-risk birth unit and 1low-risk birth unit)	Mother-newborn pairs	139 from LRBU 170 from HRBU 309 overall	24.80 (LRBU) 26.19 (HRBU) overall range 15–42	309/309 (100)
**Frezzolini, 2010**	Italy	Case-control	Not provided	Laboratory of immunology and allergology unit	Chronic urticaria patients, atopic patients, healthy controls	57 chronic urticaria patients, 22 atopic patients, 20 healthy controls	Chronic urticaria patients: 42 (11) 24–54 Not provided for controls	Chronic urticaria patients: 49/57 (86.0) Not provided for controls
**Garcia, 1997**	Spain	Case-control	Not provided	1 hospital	Cases: patients with urticaria, angioedema, or anaphylaxis; controls: healthy blood donors	61 cases, 51 controls	47 21–72 for cases, 41 18–65 for controls	39/61 (63.9) for cases, 22/47 (46.8) for controls
**García-Palacios, 1996**	Spain	Cross-Sectional	Not provided	1 hospital laboratory	Randomly selected adults showing no clinical suspicion of anisakidosis	1,008	Not provided	Not provided
**Garcia-Perez, 2015**	Spain	Case-control	2010–2013	1 hospital	gastric cancer patients, healthy controls	47 cases, 47 controls	70 (48–92) for cases, 65 (46–83) for controls	24/47 (51.1) for cases, 21/47 (44.7) for controls
**Gomez, 1998**	Spain	Case-control	1989–1996	1 allergy unit	Cases with eosiniphilic gastroenteritis, controls without digestive disorder, controls with digestive disorder different from eosinophilic gastroenteritis	10 cases, 149 controls without digestive disorder, 10 controls with digestive disorder different from eosinophilic gastroenteritis	50 for cases, not provided for controls	4/10 (40.0) for cases, not provided for controls
**González de Olano, 2007**	Spain	Cross-sectional	2003–2005	1 Allergy unit	adults with mastocytosis, children with mastocytosis, controls	163 adults with mastocytosis, 47 children with mastocytosis, 50 controls	43 (median) (18–75) for adults with mastocytosis8 (median) (0.6–14) for children with mastocytosis, Range 2–70 for controls	88/163 (54.0) for adults with mastocytosis 17/47 (36.2) for children with mastocytosis, not provided for controls
**Gonzalez Munoz, 2005**	Spain	Cross-sectional	Not provided	1 Department of Immunology	consecutive patients divided into *Anisakis* sensitized (symptoms + IgE+), chronic urticaria/abdominal pain unrelated to fish ingestion	88 overall (37 *Anisakis* sensitized, 51 with chronic urticaria/abdominal pain unrelated to fish ingestion)	median 34 (IQR = 28–48)	60/88 (68.2)
**Guillén-Bueno, 1999**	Spain	Cross-sectional	Not provided	1 hospital (gastroenterology Service)	Crohn’s disease patients, random controls	73 cases, 251 controls	35.1 (12.2) 15–73	42/73 (57.5)
**Gutierrez, 2002**	Spain	Cross-sectional	1996–1997	1 hospital (gastroenterology Service)	Gastrointestinal diseases patients, patients with digestive haemorrhaging, patients with Crohn’s disease, patients with digestive cancer, patients with appendicitis.	57 gastrointestinal diseases patients, 19 patients with digestive haemorrhaging, 30 patients with Crohn’s disease, 4 patients with digestive cancer 5 patients with appendicitis.	42.4 (17.6) gastrointestinal diseases patients, 54.3 (15.9) patients with digestive haemorrhaging, 37.2 (11.5) patients with Crohn’s disease, 71.3 (5.7) patients with digestive cancer, 24.8 (4.7) patients with appendicitis.	Not provided
**Heffler, 2016**	Italy	Cross-sectional	2010–2012	1 Allergy unit	Consecutive allergic patients	3,419	34.3 3–88	2,114/3,419 (61.8)
**Kim, 2011**	South Korea	Cross-sectional	Not provided	3 hospitals laboratories	Non allergic patients admitted for health examinations	498	from teens to 98	269/498 (54.0)
**Kimura, 1999**	Japan	Cross-sectional	1994–1997	Various laboratories throughout Japan	Allergic patients	2,108	Not provided	Not provided
**Lin, 2012**	Norway	Cross-sectional	Not provided	1 university hospital, allergy laboratory	blood donors, suspected allergy patients	100 blood donors, 798 suspected allergy patients	Not provided	Not provided
**Lin, 2014**		Cross-sectional	Not provided	1 university hospital, allergy laboratory	Blood donors, patient with total IgE levels ≥1000 kU/L	993 blood donors, 414 patient with total IgE levels ≥1000 kU/l	Not provided	Not provided
**Mazzucco,** **2012**	Italy	Cross-sectional	2009	1 hospital laboratory	Fishing industry workers	94	42.1 (12)	16/94 (17.0)
**Mladineo, 2014**	Croatia	Cross-sectional	2010–2011	1 county secondary healthcare provider Medicine-biochemical Laboratory	Unpaid randomly selected volunteer healthy subjects	500	58.1	242 (48.4)
**Montoro, 1997**	Spain	Cross-sectional	1995	1 hospital immunology and allergy service	Acute recidivous urticaria patients who usually eat fish or other seafood	25	39.3 (19.8) 11–77	16/25 (64.0)
**Nieuwenhuizen,2006**	South Africa	Cross-sectional	Not provided	1 laboratory	Fishing industry workers	578	Not provided	Not provided
**Pascual, 1996**	Spain	Cross-sectional	Not provided	1 laboratory	Patients with increased levels of serum total IgE	73	Not provided	Not provided
**Puente, 2008**	Spain	Cross-sectional	Not provided	1 laboratory	Allergic residents of Madrid, non-allergic subjects divided in patients with non-digestive non-allergic pathologies unrelated to anisakiosis and healthy residents of Madrid	86 allergic residents of Madrid, 314 non-allergic subjects divided in 50 patients with non-digestive non-allergic pathologies unrelated to anisakiosis, and 264 healthy residents of Madrid	Not provided for allergic residents of Madrid; 57.6 (20–85) for patients with non-digestive non-allergic pathologies unrelated to anisakiosis, 32.9 (18–65) for healthy residents of Madrid	Not provided
**Purello-D’Ambrosio, 2000**	Italy	Cross-sectional	Not provided	1 Laboratory	males in daily contact with fish, non atopic healthy males	28 males in daily contact with fish, 15 non atopic healthy males	30.6 (18–48) for males in daily contact with fish, 32.5 (20–44) for non atopic healthy males	0 (0.0%)
**Rodriguez, 2000**	Spain	Cross-sectional	Not provided	1 allergology clinic	Drug allergy patients	53	48.0 (16.7) 17–75	36/53 (67.9)
**Toro, 2004**	Spain	Cross-sectional	1998	1 hospital	Dyspeptic patients	174	49.3 (15.1) 21–80	83/173 (48.0)
**Uga, 1996**	Indonesia	Cross-sectional	1992–1993	1 hospital	Hospital visitors for diarrhea or routine check-ups	244	35 1–80	120/244 (49.2)
**Valinas, 2001**	Spain	Cross-sectional	Not provided	1 laboratory	Normal unpaid volunteer healthy blood donors	2,801	Not provided	Not provided
**Ventura, 2013**	Italy	Cross-sectional	Not provided	1 allergology unit	Adult allergic patients	919	17–83	622/919 (67.7)

Thirty-four studies followed a cross-sectional design, while the remaining ones (n = 7) were designed as case-control study. Twenty-two studies were made on symptomatic allergic population and among them only one specifically enrolled children (Bernardini, 2000) [39]. Three studies included only occupationally exposed population working in the fishing industry (Purello-D’Ambrosio 2000 [40], Nieuwenhuizen N 2006 [5], Mazzucco 2012 [4]), while Abattouy 2013 [41] included both inhabitants and fish workers from two coastal cities in Morocco.

Quality scores ranged from a minimum of 5 (Kimura, 1999) [20] to a maximum of 13 (Anadon 2010 [36], Mladineo, 2014 [42]) up to 14 points scale. Thirty-eight studies were considered of medium-high quality being rated 7 or more, including five studies scoring 10 and three studies scoring 11. Four studies were excluded from quality assessment since only respective abstracts were available (Estrada Rodriguez 1997 [43], Pascual 1996 [44], Rodriguez 2000 [45], Uga 1996 [46]). Extended evaluations on each items analysed for the critical appraisal are in **S2 Table**.

Data on prevalence, according to different study samples and the diagnostic tests applied, are shown in **Table 3**.

**Table 3:** Prevalence of *Anisakis* sensitization according to different study samples and diagnostic tests.

Author, year, (reference)	Sample characteristics	Sample size (n) age range (mean, SD)	Skin Prick Tests % (n), >*3 mm threshold*	ELISA/ImmunoCAP % (n), *threshold*	Other tests / criteria
**General asymptomatic population (15 studies)**					
Abattouy, 2013	Random samples	**333**	**-**	**5.1%**	**-**
Del Rey Moreno, 2006	Healthy blood donors	**77**	**-**	**22.1%** (n = 17)	Immunoblot **67.5%** recognized antigens of *A*. *simplex*
Figueiredo, 2013	Healthy military	**67**	**-**	**20.9%** (n = 14)	**-**
Frezzolini, 2010	Healthy subjects	**20**	**10.0%**	**10.0%**	**0.0%** CD63 BAT
Garcia, 1997	Healthy blood donors	**51**	**19.6%** (n = 10)	**27.4%**	Immunoblot ^**1**^ **75.0%** type 4 **1.9%** (n = 1) type 1 **15.0%** type 3
García-Palacios, 1996	Random sera	**1,008**	**-**	**6.0%** (n = 61)	**-**
Garcia-Perez, 2015	Healthy controls	**47**	**-**	**6.4%** IgA1, rAni s 1; **10.6%** IgA1, rAni s 5	**-**
Guillén-Bueno, 1999	Asymptomatic adults	**251**	**-**	**18.3%**	Immunoblot E17, 50-70-250 kDa eosinophilia, leukocytosis
Lin, 2012	Blood donors	**100**	**-**	**2.0%** ImmunoCAP > 0.35 kU/L	**-**
Lin, 2014	Blood donors	**993**	**-**	**0.4%** ImmunoCAP (**0.0%** ELISA with rAni s 1 and rAni s 7)	Immunoblot 40–100 kDa (weaker bands)
Mladineo, 2014	Random healthy	**500**	**-**	**2.0%** indirect ELISA Ani s 1 s 7	**-**
Puente, 2008	Healthy residents	**264** (18–65 years)	**-**	**11.7%** UA3 Ani s 7	**-**
Purello-D’Ambrosio, 2000	Healthy donors not occupationally exposed	**15**	**6.6%** (n = 1)	**0.0%** (n = 0) RAST	**-**
Valinas, 2001	Healthy blood donors	**2,801**	**-**	**0.4%** UA3	**-**
Ventura, 2013	Healthy controls	**187**	**16.0%**	**-**	**-**
**Occupationally exposed population, symptomatic and asymptomatic (3 studies)**					
Mazzucco, 2012	94 workers in fisheries sector: fishmongers (n = 21), fish industry emplooyees (n = 35), Fishermen/sailors (n = 38)	**94**	**-**	**11.7%** (n = 11) UniCAP-100	**-**
Nieuwenhuizen, 2006	workers employed in 2 large fish-processing workplaces in the Western Cape province of South Africa	**578**	**8.0%** (n = 46)	**-**	**-**
Purello-D’Ambrosio, 2000	Fishermen/fishmongers occupationally exposed group	**28**	**46.4%** **(**n = 13)	RAST **50.0%** (n = 14)	**-**
**Symptomatic population with allergies to any kind of allergen (24 studies)**					
Anadon, 2010	Food allergic; controls non food-related allergic	**493** food allergic; **25** controls non food-related allergic.	**-**	CAP-FEIA: **52.7%** (n = 195 true positive + 65 false positive) 3 false negative; ELISA rAni s 1 s 7: **40.2%** (n = 198) 0 false positive 0 false negative	**-**
Añíbarro, 2007	Food allergic	**436**	**12.4%** ^**2**^	**12.4%** ^**2**^	**-**
Asero, 2009	Food allergic	**1,110** (12–79 years)	**-**	**-**	**0.3%** prevalence of systemic reactions/ anaphylaxis
Bernardini, 2000	Suspect allergy	**805** (0.5–17.6 years)	**6.1%** (n = 49)	**-**	**-**
Caballero, 2012	**Sample A**: suspect allergy other than fish related; **sample B**: *Anisakis* allergic patients (anaphylaxis, angioedema, urticaria or gastrointestinal symptoms few hours after eating undercooked fish)	Sample A: **99;** sample B: **35**	Sample A: **18.0%;** sample B: **100%**	ImmunoCAP: sample A: **17.0%**; sample B: **100%**	Immunoblot rAni s 1,3,5,9,10: sample A: **15.0%**; sample B: **100%**
Consortium AAITO-IFIACI *Anisakis*, 2011	Suspect allergy	**10,570**	**4.5%** (n = 474)	**-**	Anamnesis + exclusion fish allergy: **0.6%** overall; **14.0%** of sensitized
Daschner, 2005	Chronic urticaria	**135**	**48.1%** (combined SPT+ and IgE+)	**52.6%** (only IgE+) **31.8%** (only IgG4)	**-**
Del Pozo, 1997	Urticaria/angioedema (AE) or anaphylaxis	**100**	**14.0%**	**22.0%** *(>0*.*7 kU/L)*	+ symptoms < 6 h after fish ingestion + exclusion other causes: 8.0% real allergy to *Anisakis*
Estrada Rodriguez, 1997	Asthmatic/urticaria	**66**	**-**	**19.7%** (n = 13)	**-**
Falcao, 2008	Relapsing acute urticaria	**200**	**16.5%**	**6.0%** *(>0*.*7 kU/L IgE);* **9.0%** *(>0*.*35 kU/L IgE)*	Combinations SPT IgE: **2.5%** *(SPT and >0*.*7 IgE);* **3.0%** *(SPT and >0*.*35 IgE);* **20.0%** *(SPT or> 0*.*7 IgE);* **22.5%** *(SPT or >0*.*35 IgE)*
Frezzolini, 2010	Chronic urticarial, atopic patients	**57** chronic urticarial; **22** atopic patients	**63.0%** chronic urticarial; **14.0%** atopic patients	**61.0%** *(> 0*.*35 kU/L)* chronic urticarial; **18.0%** atopic patients	CD63 BAT **67.0%** combined **75%** chronic urticarial; **0.0%** atopic patients
Garcia, 1997	Subjects with IgE against *Anisakis* divided into: **allergic** (anamnesis, the time interval <4 hours between the ingestion of fish and the onset of the reaction, and the exclusion of other causes of allergy); **non allergic** (had not eaten any fish 12 hours before the onset of the symptoms); **doubtful** (who did not remember the previous ingestion of fish or for whom the interval between ingestion and onset of symptoms was between 4 and 12 hours)	**61** overall (**25** allergic; **16** non allergic; **20** doubtful)	**92.0%** allergic; **50.0%** non allergic; **70.0%** doubtful	CAP-radioimmunoassay 100% ^3^ overall; **100%** ^3^ allergic; **100%** ^3^ non allergic; **100%** ^3^ doubtful	Immunoblot ^1^: allergic: **80.0%** type 1 pattern, **8.0%** type 3; non allergic: **12.5%** (n = 2) type 1, **56.3%** (n = 9) type 4, **19.0%** type 3; doubtful: **40.0%** type 1, **35.0%** type 3
Gomez, 1998	Suspected allergy	**147**	**10.0%** ^**2**^	**10.0%** ^**2**^	**-**
González de Olano, 2007	Mastocytosis: adults (18–65 years); children (7 months-14 years)	**163** adults; **47** children	**-**	**26.9%** (n = 44) adults; **0.0%** (n = 0) children	symptoms referred **13.6%** (n = 6) adults; **0.0%** (n = 0) children
Gonzalez Munoz, 2005	Suspect allergy subdivided into: *Anisakis* allergy; chronic urticaria or abdominal pain unrelated to fish ingestion; healthy controls	**88** overall; **37** *Anisakis* allergy; **51** chronic urticaria or abdominal pain unrelated to fish ingestion; **12** healthy controls	**-**	**42.0%** (n = 37) had a clinical history of *A*. *simplex* allergy confirmed by IgE+	CD63 BAT *Anisakis*+ vs *Anisakis*- and *Anisakis* + vs healthy controls, the cutoff for a positive basophil activation test was 21% (specificity = 96%, sensitivity = 100%), and 16% (sensitivity and specificity of 100%) respectively
Heffler, 2016	Allergic clinic outpatients	**3,419**	**15.0%**	**-**	**0.8%** + allergic symptoms after raw fish
Kimura, 1999	Urticaria or food allergy	**2,108**	**-**	**29.8**% (n = 629) *(>0*.*7 kU/L IgE)*	**-**
Lin, 2012	Serum samples from Allergy laboratory: **sample A** without any additional information on analytical results; **sample B** Phadiatop+ subjects	**600** sample A; **198** sample B	**-**	ImmunoCAP *(> 0*.*35 kU/L);* **2.2%** sample A; **6.6%** sample B	**-**
Lin, 2014	Subjects with total IgE levels ≥1000 kU/L	**414**	**-**	**16.2%** (**0.2%** ELISA with rAni s 1 and rAni s 7)	Immunoblot five bands ranging between 40 and 100 kDa to *A*. *simplex* CE
Montoro, 1997	Patients with acute recidivous urticaria who usually eat fish or other seafood.	**25**	**64.0%** (n = 16)	**76.0%** (n = 19)	Immunoblot **56.0%** (14 of the 25) tested sera showed the characteristic band at 49.8–80 kDa compared to the E17 reference serum. Most of the sera showed a common immunorecognition pattern with a group of bands at 200–80 kDa
Pascual, 1996	Patients with increased levels of serum total IgE divided into: shellfish allergy; fish allergy; probable parasitic disease; respiratory allergy	**73** overall; **16** shellfish allergy; **20** fish allergy; **17** probable parasitic disease; **20** respiratory allergy	**-**	**56.2%** (n = 41) overall; **81.3%** shellfish allergy; **40.0%** fish allergy; **58.8%** probable parasitic disease; **50.0%** respiratory allergy	**-**
Puente, 2008	Allergic residents with negative skin prick test to *Anisakis*	**86**	**0.0%** ^3^	**3.5%** UA3 Ani s 7	**-**
Rodriguez, 2000	Drug allergic patients	**53**	**54.7%** (n = 29)	**-**	**-**
Ventura, 2013	Chronic urticaria	**213**	**49.7%**	**-**	**-**
**Hospital presenting patients for any reason (5 studies)**					
Andreu-Ballester, 2008	Non appendectomized controls presenting at emergency department	**80**	**-**	**1.3%** IgG+; **7.5%** IgM+; **3.8%** IgA+; **5.0%** IgE+	**-**
Falcao, 2008	Controls selected for programmed orthopaedic, maxillofacial, or general surgery	**200** (6–18 years)	**5.5%**	**1.5%** *(>0*.*7 kU/L IgE);* **3.0%** *(>0*.*35 kU/L IgE)*	Combinations SPT ± IgE: **0.5%** *(SPT+ and >0*.*7 kU/L IgE);* **1.5%** *(SPT+ and >0*.*35 kU/L IgE);* **6.5%** *(SPT+ or > 0*.*7 kU/L IgE);* **7.0%** *(SPT or >0*.*35 kU/L IgE)*
Kim 2011	Subjects presenting at hospital for routine controls	**498**	**-**	**5.0%** larval *Anisakis* crude extract; **6.6%** *excretory-secretory proteins*	Immunoblot A specific protein band of 130 kDa was detected from 10 patients with western blot analysis against crude extract and excretory-secretory proteins among those who showed positive results by ELISA
Puente, 2008	Non-digestive nonallergic pathologies unrelated to anisakiosis	**50**	**-**	**16.0%** UA3 Ani s 7	-
Uga, 1996	Diarrhea /routine check-up without symptoms	**244**	**-**	**11.0%**	**-**
**Patients with digestive system disorders (6 studies)**					
Andreu-Ballester,2008	Cases appendectomized	**80**	**-**	**2.5%** IgG+; **2.5%** IgM+; **1.3%** IgA+; **2.5%** IgE+;	**-**
Garcia-Perez, 2015	Cases gastrointestinal cancer	**47**	**-**	**38.3%** IgA1+, rAni s 1, **42.6%** IgA1+, rAni s 5	**-**
Gomez, 1998	**Sample A**: eosinophilic gastroenteritis; **sample B**: digestive disorder different from eosinophilic gastroenteritis	Sample A: **10**; Sample B: **10**	Sample A: **80.0%**^3^ Sample B: **10.0%**^3^	Sample A: **80.0%**^3^; Sample B: **10.0%**^3^	**-**
Guillen Bueno, 1999	Crohn disease	**73**	**-**	**29.0%** specific total Ig (G+M+A) **44.0%** IgG+; **18.0%** IgM+; **53.0%** IgA+	Immunoblot: *human anisakidosis reference serum (E17); 50 and 250 kDa*, *with a band of about 70 kDa*
Gutierrez, 2002	19 digestive Haemorrhaging; 30 Crohn’s disease; 4 digestive cancer; 5 appendicitis	**57** (42.38 ± 17.60 years)	**-**	**Crude Extract:** Igs-CE **89.4%**; IgG-CE **75.4%**; IgM- CE **26.3%**; IgA- CE **63.1%**; IgE- CE **14.0%**; **Excretory- Secretory antigens:** Igs- ES **49.1%**; IgG- ES **57.8%**; IgM- ES **22.8%**; IgA- ES **57.8%**	Immunoblot **24.0%** *and* **48.0%** *of sera from patients with symptoms of Crohn’s disease and digestive haemorrhaging*, *respectively*, *showed a positive immunorecognition pattern of CE antigen*.
Toro, 2004	Dyspeptic symptoms	**174**	**-**	**13.8%** (n = 24) IgE anti Ani s 1	**-**
**Post-partum women (1 study)**					
Figueiredo, 2015	170 from high-risk birth unit and 139 from a low-risk birth unit	**309**	**-**	**19.4%** (n = 60) IgG+	**-**

^1^ Pattern types: type 1: group of several bands of medium molecular weight (MW) (30 to 50 kd) and others of low MW (14 to 30 kd); type 2: two or more bands of medium MW; type 3: only one band of medium MW (about 40 kd); type 4: negative blot without any band.

^2^ It is not specified whether each subject was tested with both IgE detection and SPT or only one diagnostic technique.

^3^ The prevalence rate is the result of an inclusion criterion of the study.

Indirect ELISA, ImmunoCAP or RAST were employed in 14 studies on general asymptomatic population; 2 studies among fishing sector workers; 18 studies including symptomatic allergic patients; 5 studies on patients admitted to hospital for any reason; 6 studies on patients with digestive disorders; and 1 study on post-partum women. Two variations of indirect ELISA, that are antigen capture ELISA [47, 35] and rAni s 1, rAni s 7 ELISA [30, 36, 42], were used, respectively, in 5 studies in the general population and in allergic patients.

Immunoblotting technique was performed along with IgE detection by means of previously cited tests in 4 studies on general asymptomatic population [30, 14, 48, 49]; 4 studies on symptomatic population with allergies to any kind of allergen [14, 30, 31,50]; 1 study on patients presenting to hospital for controls [51] and 2 studies on patients with digestive system disorders [32, 49].

General asymptomatic population was investigated for *Anisakis* sensitization through SPTs assessment in 4 studies (in 1 case it was the only diagnostic criterion employed [52]; in the remaining 3 studies, SPTs were performed along with other IgE detection techniques or CD63 BAT) [14, 38,40].

Occupationally exposed workers were assessed for *Anisakis* sensitization by SPTs alone in 1 study [5], and by both SPTs and RAST in another one [40].

Cutaneous reactivity to *Anisakis* extract among allergic patients was also evaluated in 15 studies, including 10 studies which performed both SPTs and IgE detection through indirect ELISA, ImmunoCAP or radioimmunoassays; SPTs and Immunoblot were present in 3 studies protocols; anamnesis of allergic symptoms correlated to fish consumption was considered along with SPTs in 4 studies; finally, Frezzolini et al. [38] added CD63 BAT to SPTs assessment.

Basophil activation test (BAT) was introduced by Gonzalez-Munoz et al. in 2005 [53] and later used by Frezzolini et al. [38] among allergic patients and healthy controls.

Falcao et al. assessed *Anisakis* sensitization considering the combination of both SPTs and ImmunoCAP positivity in allergic patients and in controls selected for surgery procedures [54]. Similarly, a combination of SPTs and IgE detection was performed by Gomez et al. among digestive disorders patients [55].

General asymptomatic population resulted sensitized to *Anisakis* in 0.4 to 27.4% of cases by means of indirect ELISA or ImmunoCAP specific IgE detection [14, 35], and between 6.6% and 19.6% of the samples by means of SPTs [14, 38, 40, 52]. *Anisakis* antigens recognition patterns were obtained by Immunoblotting assays in 25% [14] to 67.5% of sera from asymptomatic general population samples [48].

Occupationally exposed workers (fishermen, fishmongers and workers of fish-processing industries) had specific IgE between 11.7% [4] and 50% of cases [40], whereas SPTs positivity ranged between 8% and 46.4% [5, 40].

Symptomatic allergic patients to any kind of allergen were found to be positive to *Anisakis* specific IgE detection between 0.0% in children with mastocytosis (González de Olano 2007)[56] to 81.3% among adults with shellfish allergy (Pascual 1996) [44]; diagnostic bands at Immunoblot were visualized in 15–56% of cases (Caballero 2012)[31] (Montoro 1997)[50]. SPT positivity among allergic individuals (14 studies) ranged from 4.5% out of 10570 suspected allergy patients (Consortium 2011) [21] to 64% among 16 patients with acute recidivous urticaria, usually eating fish or other seafood (Montoro 1997) [50]. In particular, chronic urticaria patients reacted to skin tests between 14% and 63% (del Pozo 1997) [57] (Frezzolini A 2010)[38]. The SPT detected also a 14% prevalence of *Anisakis* positivity among atopic subjects (Frezzolini A 2010) [38], while it estimated a positivity ranging from 4.5% to 15% in patients presenting to allergological clinics to deal with suspected allergy (Consortium 2011) [21] (Heffler E 2016) [58]. When considering also anamnestic criteria (symptoms after fish eating), allergy to *Anisakis* was found between in 0.0–14.0% of patients [21, 56–58].

Sensitization rates in 5 study-samples selected from hospital-admitted subjects varied according to different criteria to define AS allergy, from 0.5% with a combination of both positive SPTs and >0.7 kU/L IgE (Falcão H 2008) [54] to 20% when IgE >0.35 kU/L were sufficient to be considered positive (Daschner A 1998) [59].

Six other studies investigated the seroprevalence of specific antibodies against *Anisakis* in patients with digestive system disorders (dyspepsia, appendicits/appendectomized, digestive haemorrage, gastric neoplasms), ranging from 1.3% positive to IgA (Andreu-Ballester JC 2008) [60] to 75.4% for IgG (Gutiérrez R 2002) [32]. Gomez et al. detected 80% of eosinophilic gastroenteritis (EG) patients positive to *Anisakis* SPTs, but only 10% among subjects who suffered from digestive disorders other than EG [55].

Positive immunorecognition pattern of *Anisakis* crude extracts (CE) antigens were found in 24% of sera from patients with symptoms of Crohn’s disease and 48% of those with digestive haemorrhaging [32].

Finally, IgG positivity was detected in 19.6% of a sample of postpartum women in Brazil (Figueiredo 2015) [61].

## Discussion

We identified 248 research articles and abstracts after searching various bibliographic databases and grey literature. Forty-one studies comprising 31,701 participants from eleven countries overall were included for qualitative synthesis. Most of the studies were set in high raw fish consuming countries, including Spain (n = 22, 6,734 participants) and Italy, where the largest study samples came from (8 studies comprising 17,059 participants). Also, 2 studies took place in Brazil and Norway, respectively, while 1 study was performed in each one of the following countries: Croatia, Indonesia, Japan, Morocco, Portugal, South Africa and South Korea. All the previous evidences support for a global spread of the investigated health subject.

Indirect ELISA and ImmunoCAP methods resulted the most common techniques used to assess *Anisakis* sensitization by far, measuring the presence of different classes of antibodies against various *Anisakis* allergens. As expected, higher hypersensitivity rates were obtained from selected samples of symptomatic, allergic subjects usually eating raw or undercooked seafood, coherently with the well-known association between *Anisakis* sensitization, urticaria/allergic symptoms and undercooked fish intake [44, 50], while prevalence rates tended to be lower if the study sample size was larger [30,62], and when diagnostic techniques were targeting fewer but more specific *Anisakis* antigens, or when setting higher positivity threshold for specific antibodies detection.

The results of the studies investigating *Anisakis* sensitization among the general asymptomatic population clearly highlighted the association between fish consumption and *Anisakis* sensitization. Particularly, the two studies with the largest sample size of random healthy subjects, investigated by SPTs and IgE detection, measured *Anisakis* responsiveness in 16% out of 187 individuals [52] and 6% out of 1,008, respectively [19]. Prevalence rates were greatly affected by *Anisakis* antigens chosen as target of diagnostic tests, with large differences between crude extracts of entire *Anisakis* larvae versus specific recombinant excretory-secretory proteins. More deeply, *Anisakis* larvae crude extracts (CE) might contain several cross-reactive allergens with other nematodes [63–65], crustaceans, insects or mites [44, 66, 67], and their use as target antigens in commercial assays, both serological (ImmunoCAP) and clinical ones (SPT), may lead to less specificity and consequent overestimation of seroprevalence.

Antigen capture ELISA, a variation of indirect ELISA developed to use recombinant antigens Ani s1 and Ani s7, has been applied by two Spanish studies, showing prevalence ranging from 0.4% out of 2,801 individuals [35] to 11.7% out of 264 adults [47]. Successively, another variation of rAni s1 and rAni s7 indirect ELISA was introduced by Anadon et al. [36] representing the most specific serum test to diagnose anisakiasis, revealing IgE in 40.2% out of 493 allergic subjects in Madrid, with respect to 52.7% positivity prevalence measured by ImmunoCAP from the same serum samples. Antigen capture ELISA with rAni s1 and rAni s7 was latter employed in a Croatian setting, determining 2% out of 500 random healthy subjects sampled from different areas with decreasing prevalence from a maximum of 3.5% among individuals living in islands (assumed as high fish consumers) to 1.5% in urban coastal areas, while a 0.0% prevalence was documented in a rural part of the country (declared to be an area of low or absent seafood intake), stressing the association between *Anisakis* sensitization and fish intake [42]. Recombinant Ani s1 and rAni s7 indirect ELISA was also used as second-step test to analyse ImmunoCAP positive sera obtained from Norwegian healthy blood donors and selected subjects with >1000 kU/L total IgE, resulting in prevalence rates of 0.0% and 0.2%, respectively, in comparison with 0.4% and 16.2% ImmunoCAP positivity rates from the same samples. It is not clear whether these findings confirm that significant part of the ImmunoCAP positive sera are false-positive due to cross-sensitization, or due to the unspecific binding of very high total IgE levels, or due to minor presence of *Anisakis* antigens other than rAni s1 and s7 [30]. Similar considerations regarding cross-reactivity issues and not univocal diagnostic criteria apply to the 24 studies performed in subjects with immune disorders or presenting to allergology units to rule out suspected allergy. Findings of these studies associated *Anisakis* sensitization to both relapsing acute [54] and chronic urticaria [47, 11]; furthermore, allergic manifestation after ingestion of contaminated raw or marinated fish were more frequent when patients were co-sensitized to house dust mites or molds according to SPTs, suggesting possible cross-reactive but clinically relevant allergens between these allergenic sources [58].

Generally, SPTs against *Anisakis* crude extracts resulted in wide ranges of positivity prevalence: the two largest studies measured 4.5% SPT+ out of 10570 suspected allergy subjects [21] and 15% SPT+ out of 3,410 allergy clinic outpatients [58], both percentages decreased to 0.6% and 0.8%, respectively, when allergic symptoms after raw fish intake was added as diagnostic criterion, suggesting how anamnesis plays an important role in pointing out real allergy versus possible cross-reactivity. SPT positivity without clinical manifestation can still be considered an alarm for future allergic reactions after contact with responsible antigens.

As for IgE detection, the largest Japanese study among 2,108 sera of urticaria or food allergy patients revealed 29.8% seroprevalence with a positivity threshold set at >0.7 kU/L, showing that patients suffering from type I allergic symptoms following ingestion of *Anisakis* parasitized fishes are more often sensitized to *Anisakis* specific allergen than to allergens of the seafood per se [20].

Detection of *Anisakis*-induced basophil activation (BAT) by flow cytometry was introduced by Gonzalez-Munoz et al. in 2005 [53]. Frezzolini et al. [38] latter on compared BAT with SPT and ImmunoCAP in diagnosing *Anisakis* sensitization among chronic urticaria patients, atopic subjects and healthy controls. All three tests had good similar sensitivity, but highest specificity of 100% was reached only by BAT supporting BAT as reliable diagnostic tool for anisakiasis, resulting in sensitization prevalence of 67% among chronic urticaria patients and 0% among healthy subjects.

Prevalence of detectable antibodies against *Anisakis* in six studies on patients with anamnesis of digestive disorders (dyspepsia, appendicitis, digestive haemorrhaging, Crohn’s disease, digestive cancer) ranged between 1.3% and 89.4% [32, 60]. However, most studies were of limited sample-size, therefore, no conclusive statement could be drawn in relation of Anisakis sensitization and the reported conditions. Largest sample included 174 dyspeptic patients showing IgE anti Ani s1 in 13.8% of cases. This finding suggests that *Anisakis* infection might be more frequent than expected, since only the most severe cases that require urgent upper endoscopy examination are being diagnosed at present, and because of confounding clinical manifestations with other conditions. Furthermore, uncooked-fish ingestion and previous gastric surgery were confirmed to be significantly associated with seropositivity for specific IgE against Ani s1 antigen by means of immunoblotting [68].

Although case-control studies alone are not sufficient to assess causality relationships, the significant higher ratio of positivity to secretory IgA1, rAni s1, or rAni s5 found by Garcia-Perez et al. in 47 gastric cancer patients as compared to 47 healthy controls (38.3% vs 6.4%, p-value <0.001 and 42.6% vs 10.6%, p-value <0.001, respectively), together with the evidence that some parasites inducing chronic inflammation may trigger cancer, and that *Anisakis* larvae have been co-localised incidentally in cases of gastro-intestinal tumours, could suggest that Anisakis infection might be a risk factor for the development of digestive tract cancer [33]. Parasites gastrointestinal lesions often mimic ulcers, so that patients diagnosed with digestive bleeding may suffer from unrecognized anisakiasis, explaining the high prevalence of specific antibodies and immunoblot bands of *Anisakis* reference serum [32]. By contrast, the transitory lower prevalence of anti-*Anisakis* specific immunoglobulins documented in 80 appendectomized patients was explained by a diminution of immune responses against pathogens caused by the resection of an area of the Gut Associated Lymphoid Tissue (GALT) [60], even if these results are questionable.

There are no definitive and clear patterns of bands obtained by immunoblotting assays testing for the presence of specific anti-*Anisakis* IgE. One possible explanation of large differences in molecular weights of the bands detected by immunoblotting may be the lack of unanimous preparation of *Anisakis* antigenic extracts and the different blotting conditions, which may vary the number of obtained proteins.

Immunoblotting assays were also used as second-step analysis to rule out cross-reactivity among selected sera which resulted in an already positive to *Anisakis* at ImmunoCAP or ELISA, with miscellaneous results [30, 31,52]. Most of tested sera were positive to *Anisakis* crude extracts at Immunoblot, but recognized patterns of bands were not univocal and not always concordant with the human anisakiasis reference serum (E1) [30, 52].

We have further analysed the studies dealing with occupationally exposed groups of fishermen, fishmongers and fishing industry workers who are in frequent contact with raw fish and consequently with *Anisakis* larvae [4,5,40]. Larger study samples resulted in lower sensitization prevalence: SPTs were positive in 8% out of 578 fish industry workers according to Nieuwenhuizen et al. [5] versus 46.4% out of only 28 fishermen/fishmongers in Purello-D’Ambrosio study [40]. Anisakis specific IgE were detected in 11.7% out of 94 fish sector workers by means of UniCAP-100 [4] and 50% out of 28 subjects by means of RAST [40], with antibody levels increasing with duration of occupational exposure. Being at higher risk for sensitization, fishing sector workers can represent ideal candidate for screening and development of better diagnostic tools with ameliorated sensitivity and specificity, to be successively extended in the general population.

Even if we did not perform a quantitative metanalysis, all studies which compared prevalence rates between random healthy subjects and suspected allergic or digestive disorders patients or occupationally exposed workers tended to show lower responsiveness in the former group. The wide heterogeneity in study samples characteristics, design, settings, diagnostic techniques and criteria to define *Anisakis* sensitization or allergy along with the lack of important information in a large number of studies prevented us to summarize data in order to perform a metanalysis of prevalence results.

Also, from our systematic review important weaknesses emerged referring to the quality of studies available from literature. In fact, most of the studies were not conducted on samples representative of the general population, as the sample size was not calculated a priori to accurately infer sensitization prevalence among the population of origin. A random sampling was never performed, being most of the studies conducted on a convenience sample and the response rate almost never reported. Moreover, in the few studies providing complete details on study population, especially random sera samples were often missing any information about subjects’ gender and age.

Importantly, not all studies specified target antigens of ELISA and UniCAP methods, giving only general information about specific anti-*Anisakis* IgE detection.

For the previous reasons, comparisons to rule out cross-reactivity influence or differences in specificity and sensitivity were not possible.

Furthermore, correlation between anisakiasis prevalence among different countries with fish parasitism rates of surrounding waters is not straightforward, as nowadays global trading makes seafood from very distant areas easily available. However, high fish consuming habits and genetic susceptibility linked to the presence of DRB1*1502-DQB1*0601 haplotype [69] could partially explain the widespread geographical variety observed [21,42,68].

Low sensitization prevalence among Norwegian blood donors and subjects with >1,000 kU/L total IgE despite frequent seafood intake can be explained by the absence of genetical susceptibility haplotype and by the consumption of mainly processed, canned, frozen and farmed Atlantic salmon (which was demonstrated not to be infected from anisakid nematodes) [30,70].

Lastly, as confirmed by several authors, Anisakis sensitization can be induced by ingestion of well-cooked contaminated fish due to thermo- and pepsin-resistant allergens [54, 59,71, 72], showing a residual allergenic activity also after specific heat treatment [73].

## Conclusion

This systematic review has highlighted the epidemiological impact of *Anisakis* as hypersensitivity aetiologic factor in the general population from several countries worldwide, also with regard to specific groups of patients and occupationally exposed subjects. We observed that hypersensitivity prevalence estimates varied widely according to geographical area, characteristics of the population studied, diagnostic criteria and laboratory assays with varying sensitivity and specificity. Our findings made us conclude that, if, on one hand, *Anisakis* represents a hidden cause of many adverse reactions after eating undercooked seafood, which are often claimed to be “fish allergy”, including chronic idiopathic urticaria, on the other hand, further studies are needed to overcome the documented misdiagnosis by improving the diagnostic approach and, consequently, to provide more affordable estimates in order to address public health interventions on populations at high risk of exposure to *Anisakis* and to tailor health services related to specific groups.

## Supporting information

S1 Database Final database of all included studies. (ZIP)

S1 Table PRISMA checklist. (DOC)

S2 Table Quality assessment of the studies included. (DOCX)

## Abbreviations

PRISMA: Preferred Reporting Items for Systematic Reviews and Meta-Analyses
AS: Anisakis
ELISA: enzyme-linked immunosorbant assay
MeSH: Medical Subject Headings
SPT: Skin prick test
RAST: Radio-Allergo-Sorbent Test
OD: optical density
BAT: Basophil activation test
EG: eosinophilic gastroenteritis
CE: crude extracts
ES: excretory-secretory
GALT: Gut Associated Lymphoid Tissue
