# Supplementary material for: Anisakis sensitization in different population groups and public health impact: A systematic review
Source: PLoS One. 2018 Sep 20;13(9):e0203671. doi: 10.1371/journal.pone.0203671 (PMC6147426; doi:10.1371/journal.pone.0203671)
Supplement: S2 Table — (DOCX) [file pone.0203671.s003.docx]

## S2 Table. Quality assessment of the studies included.

| **Author, year, and reference** | **Representativeness of sample** | **Participant recruitment** | **Sample size** | **Description of participants and setting** | **Response rate, %^†^** | **Objective, reliable measurement of Anisakis simplex hypersensitivity** | **Was the condition measured reliably?** | **Was there appropriate statistical analysis?** | **Are all important confounding factors/ subgroups/differences identified and accounted for?** | **Were subpopulations identified using objective criteria?** | | **Quality indicators met** |
| --- | --- | --- | --- | --- | --- | --- | --- | --- | --- | --- | --- | --- |
| Abattouy, 2013 | 1 | 1 | 1 | 1 | 0 | 1 | 1 | 1 | 1 | 1 | | **9** |
| Anadon, 2010 | 1 | 0 | 1 | 1 | 0 | 3 | 2 | 1 | 1 | 1 | | **11** |
| Andreu-Ballester, 2008 | 1 | 1 | 0 | 1 | 0 | 1 | 1 | 1 | 0 | 0 | | **6** |
| Añíbarro, 2007 | 1 | 0 | 1 | 1 | 0 | 3 | 1 | 0 | 0 | 0 | | **7** |
| Asero, 2009 | 1 | 0 | 1 | 1 | 0 | 3 | 1 | 1 | 1 | 1 | | **10** |
| Bernardini, 2000 | 1 | 0 | 1 | 1 | 0 | 2 | 1 | 1 | 1 | 1 | | **9** |
| Caballero, 2012 | 1 | 0 | 0 | 1 | 0 | 2 | 2 | 1 | 1 | 1 | | **9** |
| Consortium, AAITO-IFIACI  Anisakis 2010 | 1 | 0 | 1 | 1 | 0 | 3 | 1 | 1 | 1 | 1 | | **10** |
| Daschner, 2005 | 1 | 0 | 0 | 1 | 0 | 1 | 1 | 1 | 1 | 1 | | **7** |
| del Pozo, 1997 | 1 | 0 | 0 | 0 | 0 | 3 | 1 | 0 | 1 | 1 | | **7** |
| Del Rey Moreno, 2006 | 1 | 1 | 0 | 1 | 0 | 2 | 1 | 1 | 1 | 1 | | **9** |
| Estrada Rodriguez, 1997 | Only abstract available | | | | | | | | | | | |
| Falcao, 2008 | 1 | 0 | 1 | 0 | 0 | 3 | 1 | 1 | 0 | 0 | **7** | |
| Figueiredo, 2013 | 0 | 0 | 0 | 1 | 0 | 3 | 1 | 1 | 0 | 0 | **6** | |
| Figueiredo, 2015 | 1 | 1 | 1 | 1 | 0 | 2 | 2 | 1 | 1 | 1 | **11** | |
| Frezzolini, 2010 | 0 | 0 | 0 | 1 | 0 | 2 | 1 | 1 | 1 | 0 | **6** | |
| Garcia,1997 | 1 | 0 | 0 | 1 | 0 | 3 | 1 | 1 | 1 | 1 | **9** | |
| Garcia-Palacios, 1996 | 1 | 1 | 1 | 0 | 0 | 2 | 2 | 1 | 0 | 0 | **8** | |
| Garcia-Perez, 2015 | 1 | 0 | 0 | 1 | 0 | 3 | 2 | 1 | 1 | 1 | **10** | |
| Gomez,1998 | 1 | 0 | 0 | 1 | 0 | 2 | 0 | 1 | 1 | 1 | **7** | |
| Gonzalez Munoz, 2005 | 1 | 0 | 0 | 1 | 0 | 2 | 1 | 1 | 0 | 0 | **6** | |
| González de Olano, 2007 | 1 | 0 | 1 | 1 | 0 | 2 | 1 | 1 | 0 | 0 | **7** | |
| Gutierrez, 2002 | 1 | 0 | 0 | 1 | 0 | 1 | 2 | 1 | 0 | 1 | **7** | |
| Guillén-Bueno, 1999 | 1 | 0 | 1 | 1 | 0 | 1 | 2 | 1 | 1 | 1 | **9** | |
| Heffler, 2016 | 1 | 0 | 1 | 1 | 1 | 2 | 1 | 1 | 1 | 1 | **10** | |
| Kim, 2011 | 1 | 0 | 1 | 1 | 0 | 1 | 1 | 0 | 1 | 1 | **7** | |
| Kimura,1999 | 0 | 0 | 1 | 0 | 0 | 2 | 1 | 1 | 0 | 0 | **5** | |
| Lin, 2012 | 1 | 0 | 1 | 0 | 0 | 1 | 2 | 1 | 1 | 1 | **8** | |
| Lin, 2014 | 1 | 0 | 1 | 0 | 0 | 1 | 2 | 1 | 1 | 1 | **8** | |
| Mazzucco, 2012 | 1 | 0 | 0 | 1 | 0 | 3 | 1 | 1 | 1 | 1 | **9** | |
| Mladineo, 2014 | 1 | 1 | 1 | 1 | 1 | 3 | 2 | 1 | 1 | 1 | **13** | |
| Montoro, 1997 | 1 | 0 | 0 | 0 | 0 | 3 | 2 | 1 | 0 | 0 | **7** | |
| Nieuwenhuize,n 2006 | 1 | 0 | 1 | 1 | 0 | 2 | 1 | 1 | 1 | 1 | **9** | |
| Pascual, 1996 | Only abstract available | | | | | | | | | | | |
| Puente, 2008 | 1 | 0 | 1 | 1 | 0 | 2 | 1 | 1 | 1 | 1 | **9** | |
| Purello-D’Ambrosio, 2000 | 1 | 0 | 0 | 1 | 0 | 2 | 1 | 1 | 1 | 1 | **8** | |
| Rodriguez, 2000 | Only abstract available | | | | | | | | | | | |
| Toro, 2014 | 1 | 0 | 0 | 1 | 0 | 2 | 2 | 1 | 1 | 1 | **9** | |
| Uga,1996 | Only abstract available | | | | | | | | | | | |
| Valinas, 2001 | 1 | 1 | 1 | 1 | 0 | 2 | 2 | 1 | 1 | 1 | **11** | |
| Ventura, 2013 | 1 | 0 | 1 | 1 | 0 | 3 | 1 | 1 | 1 | 1 | **10** | |
